# A pilot meta-analysis on self-reported efficacy of neurofeedback for adolescents and adults with ADHD

**DOI:** 10.1038/s41598-022-14220-y

**Published:** 2022-06-15

**Authors:** Hsin-Yi Fan, Cheuk-Kwan Sun, Yu-Shian Cheng, Weilun Chung, Ruu‐Fen Tzang, Hsien‐Jane Chiu, Chun-Ning Ho, Kuo-Chuan Hung

**Affiliations:** 1 Department of Psychiatry, Tsyr-Huey Mental Hospital, Kaohsiung Jen-Ais Home, Taiwan; 2 Department of Emergency Medicine, E-Da Hospital, Kaohsiung City, Taiwan; 3 School of Medicine for International Students, I-Shou University, Kaohsiung City, Taiwan; 4 Institute of Biomedical Sciences, National Sun Yat-Sen University, Kaohsiung City, Taiwan; 5 Department of Psychiatry, Mackay Memorial Hospital, Taipei City, Taiwan; 6 Taoyuan Psychiatric Center, Ministry of Health and Welfare, Taoyuan City, Taiwan; 7 Institute of Hospital and Health Care Administration, National Yang-Ming University, Taipei City, Taiwan; 8 Department of Anesthesiology, Chi Mei Medical Center, No. 901, ChungHwa Road, YungKung Dist, Tainan, 71004 Taiwan

**Keywords:** Medical research, Molecular medicine

## Abstract

Self-reported effectiveness of electroencephalogram-based neurofeedba*ck* (*EEG-NF*) against the core symptoms of attention-deficit hyperactivity disorder (ADHD) in adolescents/adults remains unclear. We searched PubMed, Embase, ClinicalKey, Cochrane CENTRAL, ScienceDirect, Web of Science, and ClinicalTrials.gov from inception to August 2021 for randomized clinical trials (RCTs) of EEG-NF with self-reported ADHD symptom ratings. Comparators included participants on waitlist/treatment as usual (TAU) or receiving other interventions. Of the 279 participants (mean age = 23.48; range: 6–60) in five eligible RCTs, 183 received EEG-NF treatment. Forest plot demonstrated no difference in inattention (SMD = −0.11, 95% CI −0.39–0.18, *p* = 0.46), total score (SMD = −0.08, 95% CI −0.36–0.2, *p* = 0.56), and hyperactivity/impulsivity (SMD = 0.01, 95% CI −0.23–0.25, *p* = 0.91) between EEG-NF and comparison groups. Nevertheless, compared with waitlist/TAU, EEG-NF showed better improvement in inattention (SMD = −0.48, 95% CI −0.9–−0.06, *p* = 0.03) but not hyperactivity/impulsivity (SMD = −0.03, 95% CI −0.45–0.38, *p* = 0.87). Follow-up 6–12 months demonstrated no difference in inattention (SMD = −0.01, 95% CI −0.41–0.38, *p* = 0.94), total score (SMD = 0.22, 95% CI −0.08–0.52, *p* = 0.15), and hyperactivity/impulsivity (SMD = −0.01, 95% CI −0.27–0.26, *p* = 0.96) between the two groups. Dropout rate also showed no difference (RR = 1.05, 95% CI 0.82–1.33, *p* = 0.72). Our results support EEG-NF for improving inattention in adolescents/young adults, although its effectiveness against hyperactivity/impulsivity remains inconclusive.

## Introduction

There are both pharmacological and non-pharmacological treatments for attention-deficit hyperactivity disorder (ADHD), a global illness with a prevalence of up to 5% among children and adolescents^1^. Although pharmacotherapy remains the mainstay of treatment for severe cases^2^, its long-term safety and efficacy remain unclear^3^. Indeed, side-effects associated with pharmacological treatment of ADHD are still an important concern^4–6^. To minimize drug-related side-effects and enhance treatment efficacy, a recent review has endorsed a combination of pharmacotherapy and cognitive behavioral therapy (CBT)^7^, despite a lack of information about its long-term outcome.

On the other hand, there has been evidence in favor of the use of electroencephalogram-based neurofeedback (EEG-NF) in the treatment of patients with ADHD^8,9^. EEG-NF involves the conversion of extracted signals of brain activities into pre-selected brain parameters in the form of a specific brain potential or frequency band (e.g., theta and beta) that are made perceivable to the participant. Such a feedback could allow neuroplasticity to improve cognition and behavior through self-regulation of one’s own brain activity^9^. EEG-NF is currently used for normalizing aberrant brain activity associated with neurocognitive disorders, enhancing cognitive performance in healthy individuals (i.e., peak performance training), and studying the role of neural oscillations in behavior and cognition^9^. Despite its development as early as in the 1930s^8^ and the first demonstration of its potential effectiveness against hyperactivity and distractibility in a child with hyperkinetic syndrome^10^, the benefits of EEG-NF in the treatment of the core symptoms of ADHD remains controversial. While some studies supported its effectiveness against the symptoms of ADHD with minimal-to-no side-effects^8,9^, another study on adult patients did not share the same findings^11^.

Current application of EEG-NF in patients with ADHD mainly focuses on enhancing beta activity or suppressing theta/beta (4–7 Hz/12–21 Hz) ratio (TBR) in central and frontal locations as high theta power, high theta/beta ratios, and/or low beta power are commonly observed in children and adults with ADHD^12–15^. Nevertheless, previous studies have demonstrated a relative reduction in beta activity with increasing age in patients with ADHD^13^. Together with the clinical observation of a diminished hyperactivity component in adult patients compared with that in children with ADHD despite persistent impulsivity in the former, a previous study has proposed an association between decreased beta activity and hyperactivity as well as that between increased theta activity and impulsivity^13^. Hence, the findings may suggest a potential difference in effectiveness of EEG-NF against the core symptoms of ADHD between children and adults. Another issue when considering the difference in effectiveness of EEG-NF against symptoms of ADHD between children and older individuals is the approach to outcome assessment; while children were mostly evaluated by proximal and probably blind observers^16,17^, adolescent and adult patients can self-report the efficacy of treatment.

Therefore, focusing on adolescents and adults, the current meta-analysis reviewed the efficacy of surface EEG-NF against the symptoms of ADHD from a patient’s perspective. We also investigated the therapeutic impact of EEG-NF on different core symptoms of ADHD (i.e., inattention, hyperactivity, and impulsivity).

## Methods

### Guidelines and registration

The current meta-analysis was conducted according to the guideline of Preferred Reporting Items for Systematic Reviews and Meta-Analyses (PRISMA) statement, and was registered at the PROSPERO international database (Number: CRD42021275952).

### Search strategy

We searched the databases of PubMed, Embase, ClinicalKey, Cochrane CENTRAL, ScienceDirect, Web of Science, and ClinicalTrials.gov from inception to August 2021. The keywords used in different databases are listed in Supplemental Table 1. No publication date or language restriction was applied. To expand our search results, the reference lists of specific review/original articles were also searched for cross-referencing.

### Inclusion and exclusion criteria

Studies meeting the following PICOS criteria were considered eligible for inclusion: (1) Population: patients who had a diagnosis of ADHD compatible with that documented in the Diagnostic and Statistical Manual of Mental Disorders (DSM) or the International Statistical Classification of Diseases and Related Health Problems (ICD); (2) Intervention: surface electroencephalography neurofeedback (EEG-NF); (3) Comparison interventions: waitlist, treatment as usual (TAU) or any interventions other than surface EEG-NF such as pharmacological treatment or cognitive training; (4) Outcome: self-reported rating scales for symptoms of ADHD; and (5) Study design: randomized controlled trials (RCTs). The exclusion criteria included: (1) animal studies, (2) unavailable information about target outcomes, and (3) non-RCTs.

### Strategies for data extraction

After removal of duplicates, two authors examined the eligibility of the articles according to the titles and abstracts. In the situation of discrepancy, the corresponding author was involved. On full-text review, an independent author selected trials for inclusion and documented reasons for exclusion of the ineligible studies. The above three authors also extracted the data of primary and secondary outcomes as well as clinical variables of interest, such as duration of follow-up, mean age, gender distribution, and number of NF sessions. When data were unavailable in the included trials, the authors of the selected studies were contacted for missing information up to two times over a month for data acquisition.

### Primary outcome, secondary outcomes, and definitions

Primary outcomes were improvements in self-reported rating scales, including sub-scales such as inattention and hyperactivity/impulsivity for symptoms of ADHD, and secondary outcomes were dropout rates.

### Assessment of risk of bias for the included studies

For each trial, two authors independently assessed the risk of bias using the criteria outlined in the Cochrane Handbook for Systematic Reviews of Interventions, including the overall risk of bias of all the included trials and the risk of bias of individual studies^18^. The risk of bias was classified as “low”, “unclear”, or “high” for the following key domains: (a) sequence generation; (b) allocation concealment; (c) blinding of participants and personnel; (d) blinding of outcome assessment; (e) incomplete outcome data; (f) selective outcome reporting; and, (g) other sources of bias. Disagreements were solved by discussion.

### Statistical analysis

Cochrane Review Manager (RevMan 5.3; Copenhagen: The Nordic Cochrane Center, The Cochrane Collaboration, 2014) was applied for data synthesis. For dichotomous outcomes, risk ratios (RRs) with 95% confidence intervals (CIs) were calculated by using a random effects model. The Mantel–Haenszel (M–H) method was used to pool dichotomous data and to compute pooled RRs with 95% CIs. As the included trials may use different scales to measure the same outcomes, standardized mean differences (SMD) with 95% CIs were calculated to estimate the effect size. The I^2^ statistic was utilized for heterogeneity assessment (low: 0% to 50%; moderate: 50% to 75%, high: 75% to 100%)^19^. To evaluate stability of the results, sensitivity analysis was conducted by removing one study each time. We examined the funnel plots when we identified 10 or more studies reporting on a particular outcome to investigate the potential of reporting and publication bias. The significance level was set at 0.05 for all analyses.

## Results

### Eligible studies and assessment tools

**Figure 1:**
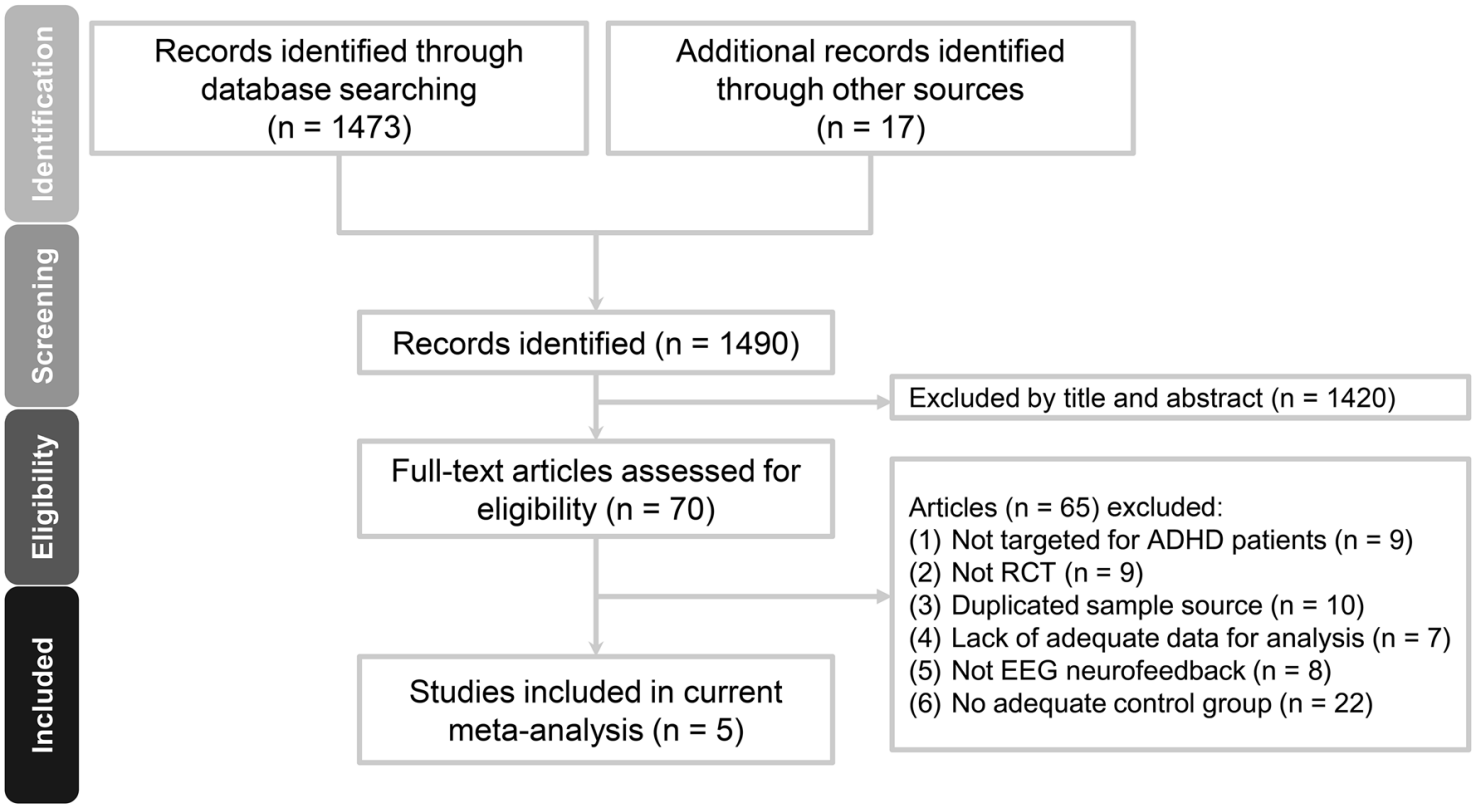
Meta-analysis flowchart for selecting eligible studies. RCT: randomized controlled trial; ADHD: attention-deficit hyperactivity disorder; EEG: electroencephalogram.

The PRISMA flowchart summarizing the process of study selection in this systematic review is shown in Fig. 1.The PRISMA checklist and the reasons for study exclusion are presented in Supplemental Tables 2 and 3, respectively. Of the 70 full-text articles assessed for eligibility, 65 were excluded because they did not meet the inclusion criteria. Finally, five articles using an RCT design were selected for the current study^11,20–23^.

**Figure 2:**
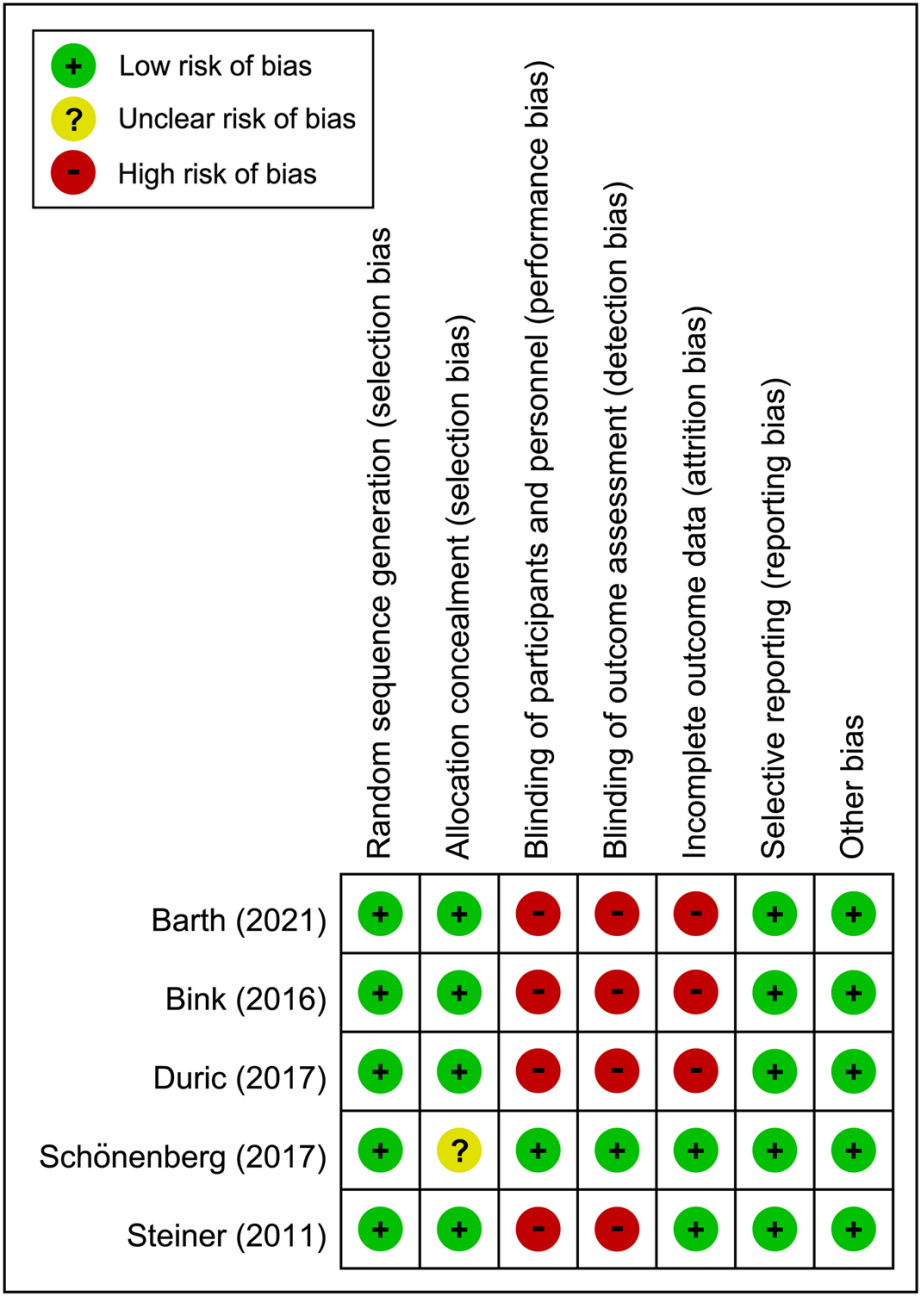
Risks of bias of individual studies.

**Figure 3:**
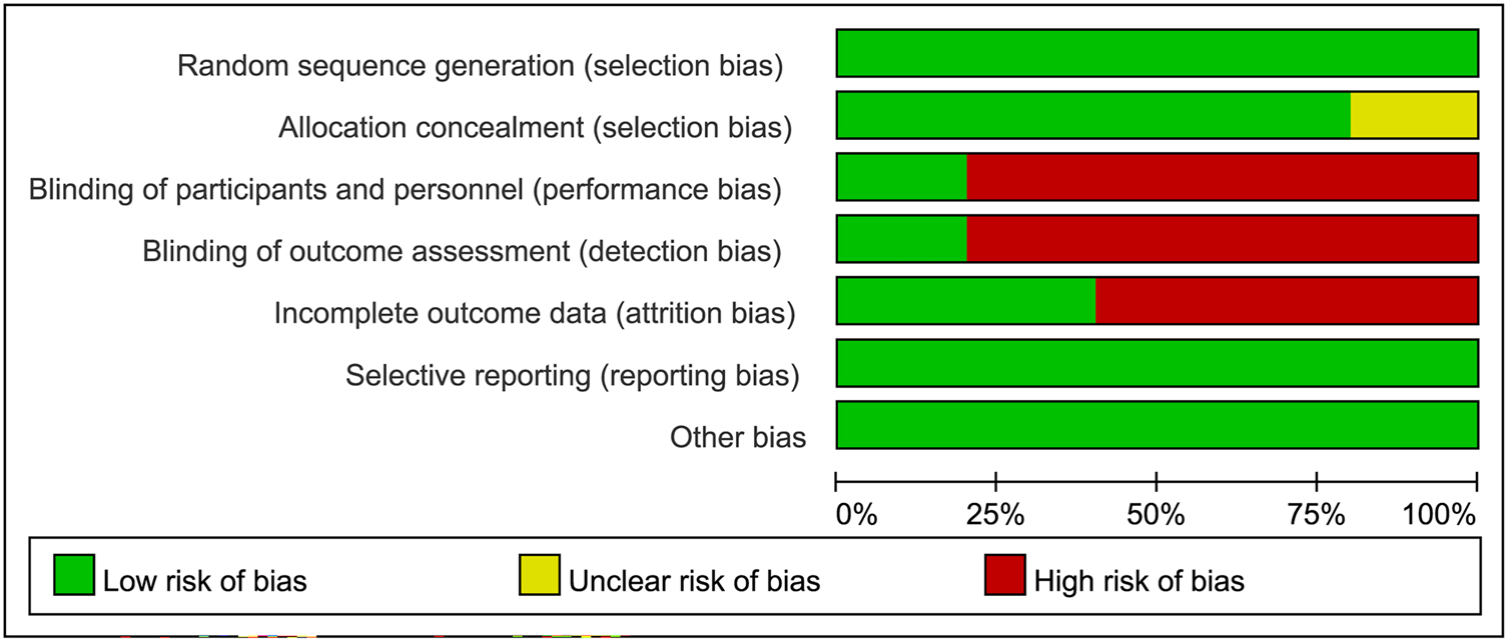
Overall risks of bias of the included studies.

The characteristics of studies are shown in Table 1. Among a total of 279 participants with a mean age of 23.48 years (range: 6–60 years) and a male prevalence of 69.5% (range: 39.1%–100.0%) included in the current study, 183 received NF treatment. All trials expect one^21^ allowed the use of psychostimulants in different proportions of participants (13.3–60%) in their study groups (Table 1). The therapeutic approaches of EEG-NF mainly comprised theta/beta (TB) and theta/sensorimotor rhythm (SMR) training programs which involve inhibiting rhythmic slow activity (theta) and rewarding the SMR/beta frequency bands. Only one study used slow cortical potential (SCP) in its EEG-NF protocol^23^. The self-report instrument used in each study is shown in Table 1. The diagnostic criteria for ADHD were mainly based on DSM-IV-TR and ICD-10 with adolescents and young adults being the main targeted groups. Most participants were allowed to receive medications except in one study^21^. Comparison group consisted of waitlist/TAU^20,22^, electromyography (EMG) biofeedback^23^, medications^21^, and sham control^11^. Of all the eligible studies, four conducted a follow-up investigation^11,20,22,23^. The post-treatment follow up duration ranged from 6 to 12 months. Focusing on the risk of bias (Figs. 2 and 3), most studies did not use double-blind design except one^11^. Taking into account the seven domains of the Cochrane Collaboration risk of bias tool for the five included trials, we found that 65.7% (23/35), 2.9% (1/35), and 31.4% (11/35) of the included studies had an overall low, unclear, and high risk of bias, respectively. None of the studies received financial support from pharmaceutical companies.

**Table 1:** Summary of characteristics of studies in the current meta-analysis.

Author (year)	Diagnosis (Criteria)	Design (Blinding)	Comparison	Session	*N*	Duration (weeks)	Outcome	IQ	Stimulant (%)	Age (range) (years)	Female (%)	Country
Barth (2021)	ADHD (DSM-IV)	RCT (No)	NF: SCP	30	26	26.64 FU: 6 M	ADHS-SB: Attention, global, hyperactivity and impulsivity	108.32	23.1	33.63 (18–56)	60.9	Germany
			EMG-biofeedback		20				25			
Schönenberg (2017)	ADHD (DSM-IV-TR)	RCT (Yes)	NF: TB	30	37	15 FU: 6 M	CAARS-self-rating: Attention, total, hyperactivity and impulsivity	N/A	16	37.8 (18–60)	44	Germany
			Sham		38				11			
Duric (2017)	Hyperkinetic disorder (ICD-10)	RCT (No)	NF: TB (theta/SMR)	30	30	12 FU: 6 M	Barkley’s Defiant Children-self-repot: Attention, total, hyperactivity	87.98	0	11.15 (6–18)	19.67	Norway
			MPH		31				100			
Bink (2016)	ADHD (DSM-IV-TR)	RCT (No)	NF: TB (theta/SMR)	37	45	25 FU: 1 year	1.ADHD rating- self-report: Inattention, H/I 2.Youth self-report: total	100.66	44	16.14 (12–24)	0	Netherlands
			TAU		26				62			
Steiner (2011)	ADHD (By physician)	RCT (No)	NF: TB	23.4	9	16 FU: N/A	CRS-R self-report: inattention, ADHD index, hyperactivity	N/A	60	12.4 (11–14)	47.8	US
			Waiting list		15							

ADHD, Attention deficit and hyperactivity disorders; ADHS-SB German ADHD self-rating scale for symptoms in adulthood; CAARS Conners’ adult ADHD rating scale; CRS-R, Conners’ Rating Scale – revised; DSM-IV Diagnostic and Statistical Manual of Mental Disorders, fourth edition; DSM-IV-TR, Diagnostic and Statistical Manual of Mental Disorders, fourth edition, text revision; EMG electromyography; FU follow up; H/I, hyperactivity/impulsivity; ICD-10 International Classification of Diseases, Tenth Revision; IQ intelligence quotient; MPH, methylphenidate; M month; N, number; N/A not available; NF, neurofeedback; RCT randomized controlled trial; SCP slow cortical potentials; SMR, Sensorimotor rhythm; TAU, treatment as usual; TB, theta/beta training; yr year.

### Impact of neurofeedback on symptoms of ADHD

**Figure 4:**
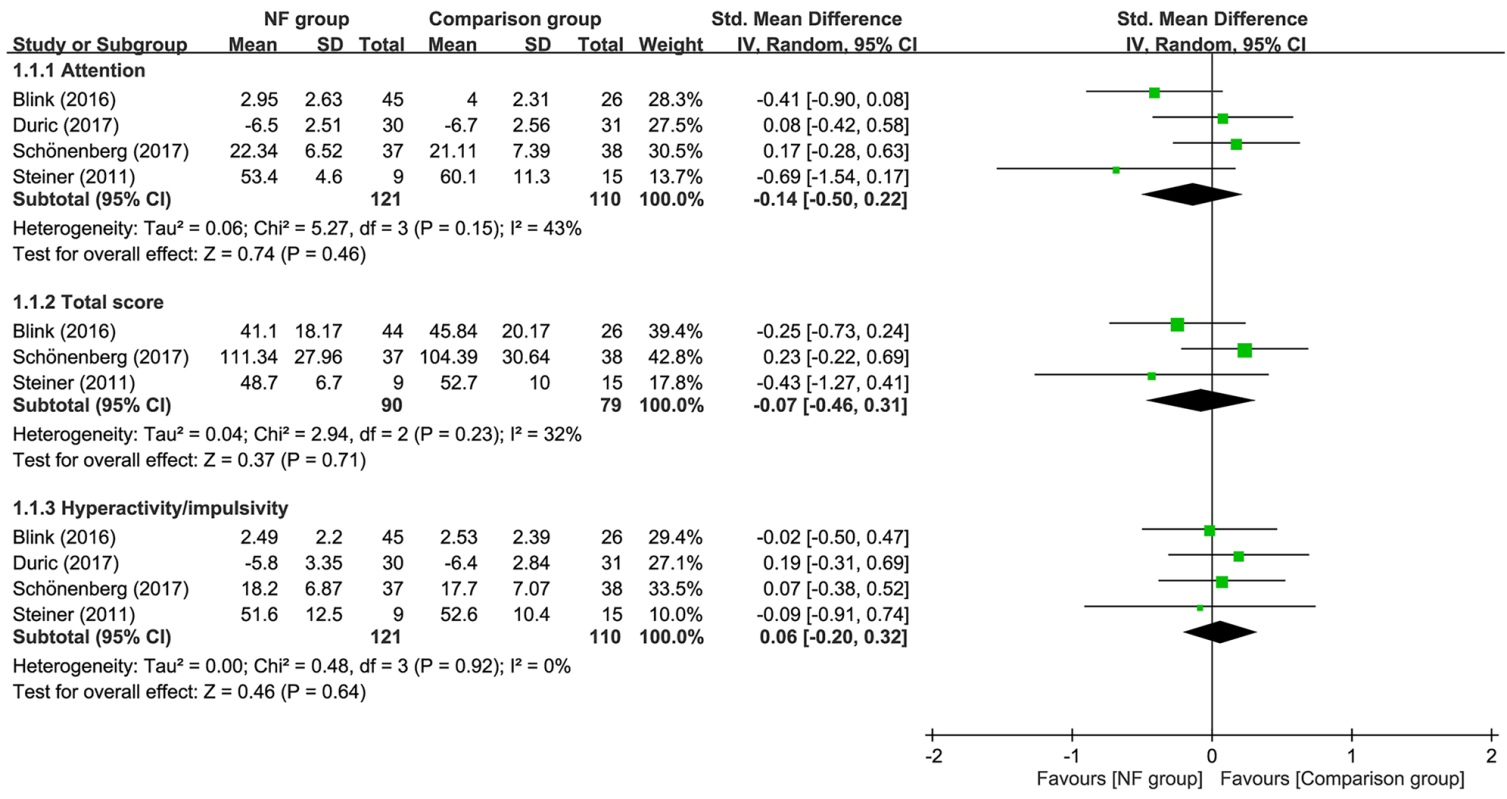
Forest plot for comparing symptoms of attention-deficit hyperactivity disorder between EEG-NF and comparison groups. CI, confidence interval; IV, inverse variance; Std, standardized.

Forest plot demonstrated that there was no difference in inattention (SMD = −0.11, 95% CI −0.39 to 0.18, *p* = 0.46; I^2^ = 25%; five trials, *n* = 277), total score (SMD = −0.08, 95% CI −0.36 to 0.2, *p* = 0.56; I^2^ = 4%; four trials, *n* = 215), and hyperactivity/impulsivity (SMD = 0.01, 95% CI −0.23 to 0.25, *p* = 0.91; I^2^ = 0%; five trials, *n* = 277) between EEG-NF and comparison groups (Fig. 4). Sensitivity analysis demonstrated no significant impact on these outcomes by omitting any trials.

### Comparison of neurofeedback with waitlist/TAU on symptoms of ADHD

**Figure 5:**
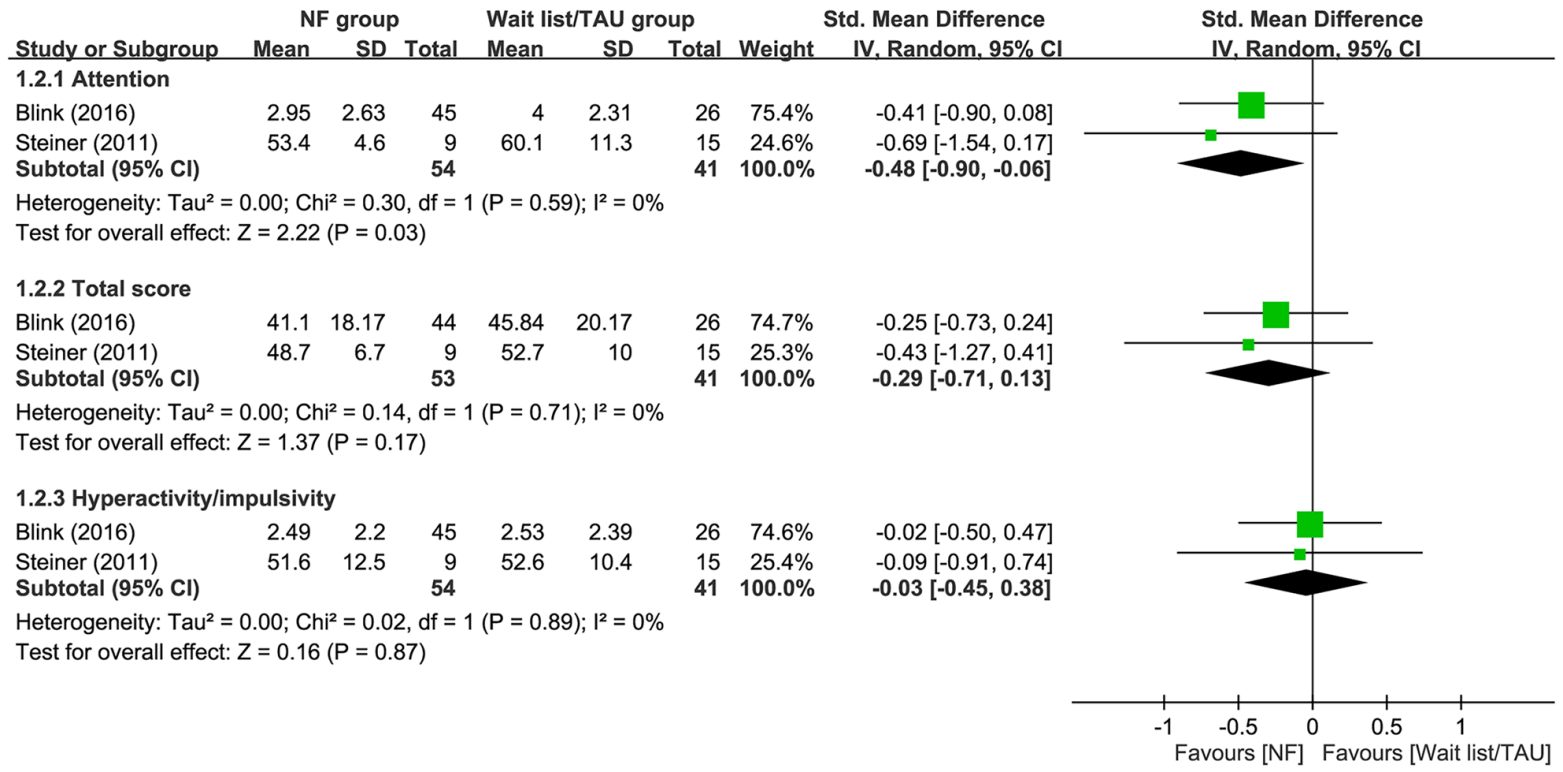
Forest plot for comparing symptoms of attention-deficit hyperactivity disorder between EEG-NF and waitlist/TAU groups. CI, confidence interval; IV, inverse variance; Std, standardized.

Our results showed that the use of EEG-NF was associated with a significantly better improvement in inattention compared to that in those on waitlist or receiving TAU (SMD = −0.48, 95% CI −0.9 to −0.06, *p* = 0.03; I^2^ = 0%; two trials, *n* = 95) (Fig. 5). However, there was no difference in total score (SMD = −0.29, 95% CI −0.71 to 0.13, *p* = 0.17; I^2^ = 0%; two trials, *n* = 94) and hyperactivity/impulsivity (SMD = −0.03, 95% CI −0.45 to 0.38, *p* = 0.87; I^2^ = 0%; two trials, *n* = 95) between patients receiving EEG-NF and those serving as waitlist/TAU controls (Fig. 5). Sensitivity analysis was not performed as only two trials were available for outcome analyses.

### Follow-up outcomes between neurofeedback and comparison groups

**Figure 6:**
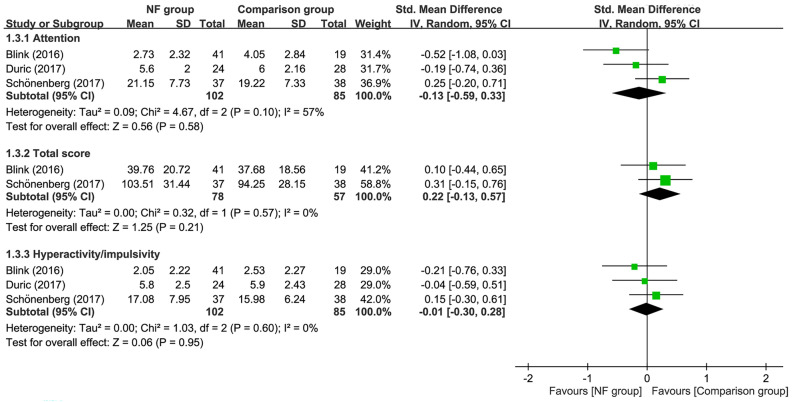
Forest plot for comparing symptoms of attention-deficit hyperactivity disorder between EEG-NF and comparison groups during 6–12 months follow-up. CI, confidence interval; IV, inverse variance; Std, standardized.

Follow-up for 6–12 months demonstrated no difference in inattention (SMD = −0.01, 95% CI −0.41 to 0.38, *p* = 0.94; I^2^ = 55%; four trials, *n* = 233), total score (SMD = 0.22, 95% CI −0.08 to 0.52, *p* = 0.15; I^2^ = 0%; three trials, *n* = 181), and hyperactivity/impulsivity (SMD = −0.01, 95% CI −0.27 to 0.26, *p* = 0.96; I^2^ = 0%; four trials, *n* = 233) between EEG-NF and comparison groups (Fig. 6). Sensitivity analysis indicated stability of the merged results of outcomes when removing one study at a time.

### Dropout rate

**Figure 7:**
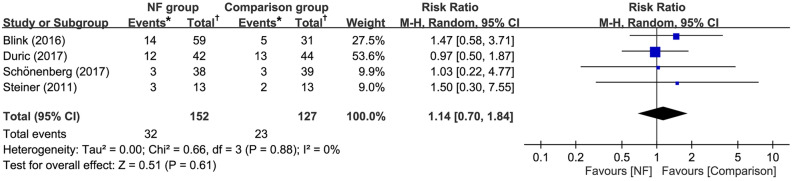
Forest plot for comparing dropout rate between EEG-NF and comparison groups. CI, confidence interval; M-H, Mantel–Haenszel. *Total number of participants in intervention groups or in comparison groups; †Number of dropout events in intervention groups or in comparison groups.

Forest plots of the five studies with a total of 330 patients (NF group, *n* = 183 vs. comparison group, *n* = 147) available for the analysis of dropout rate showed no significant difference between EEG-NF and comparison groups (RR = 1.05, 95% CI 0.82 to 1.33, *p* = 0.72; I^2^ = 0%) (Fig. 7). Besides, Sensitivity analysis demonstrated no significant effect on this outcome by omitting certain trials.

## Discussion

To our best knowledge, this is the first meta-analysis to focus on the efficacy of surface EEG-NF against the symptoms of ADHD based on subjective reports from patients’ perspective. Although there were many previous meta-analyses investigating the therapeutic effects of surface EEG-NF on ADHD symptoms^16,17,24–28^, they mainly used outcome measurements from teachers’ or parents’ observations. In fact, discrepancies in treatment outcomes between different observers were reported in previous meta-analyses^16,17^ with a reduction in effect size (ES) of EEG-NF up to 24% to 50% being reported in patients with ADHD when comparing the rating by most proximal (i.e., the closest) evaluators with that by probably blind observers^16,17^. Therefore, it is important to understand the patients’ subjective feelings about the effectiveness of surface EEG-NF, especially for adolescents and adults who have a better self-awareness of ADHD symptoms than that in children, because their views may be different from those of their parents or teachers^29^. Moreover, motivation to participate in a treatment program is also an important factor influencing the therapeutic outcome, especially for adolescents^30^. From our research, it is surprising that we were only able to identify five RCTs which included self-reported rating scales in their outcome measurements^11,20–23^, even though there were more than 20 RCTs investigating the efficacy of surface EEG-NF in patients with ADHD^16,17^. It seems that therapeutic effects of surface EEG-NF from patients’ own perspective were largely ignored in the previous RCT studies, perhaps partly due to a poor awareness of the patients’ own attentional status among young children^31^. Due to the relative paucity of previous RCTs focusing on self-reported efficacy of surface EEG-NF, our results were only preliminary. Our findings showed a significantly better improvement in inattention among adolescents and young adults with ADHD receiving surface EEG-NF (SMD = −0.48, *p* = 0.03) than that in those in the waitlist/TAU group. However, surface EEG-NF was not significantly more effective for improving symptoms of ADHD when compared with a mixed group of comparators (i.e., TAU/waitlist, methylphenidate or sham control).

Previous meta-analyses failed to demonstrate a consistent finding regarding the efficacy of surface EEG-NF between most proximal (e.g., parents) and probably blind (e.g., teachers) evaluators. A meta-analysis which included both active and non-active treatments in the comparison group showed a superior effectiveness of surface EEG-NF against the symptoms of ADHD only from the observations of most proximal evaluators (ES: 0.36, *p* = 0.009) but not from the evaluation by blind evaluators (ES: 0.15, *p* = 0.20)^17^. Our study result, which failed to show significantly better therapeutic effects of surface EEG-NF than other active interventions, is consistent with the observation from probably blind evaluators in that meta-analysis^17^. Moreover, apart from the impact of evaluators on treatment outcomes of surface EEG-NF^16,17^, age could also be an important confounder. Because children may be less aware of their attentional problems, they may benefit more from EEG-NF than adults based on the theories of operant conditioning^32^ and brain plasticity^33^. Therefore, the inclusion of much older age groups (mean age: 23.48 years) in our meta-analysis than those in previous meta-analyses focusing on children^16,17^, may also contribute to the relatively weak efficacy of NF in our study. Indeed, previous studies on exclusively adult patients demonstrated that, although surface EEG-NF was effective for alleviating symptoms of ADHD, it offered no additional therapeutic benefit than that in sham controls or those undergoing meta-cognitive training or EMG biofeedback ^11,23^. Therefore, further studies are warranted to investigate the effects of age and patients’ subjective perception on the efficacy of surface EEG-NF in adolescents and adults.

Despite the failure of our preliminary findings to show a better effectiveness of EEG-NF than that of other active interventions, our subgroup analysis found a significantly better improvement in inattention in patients receiving surface EEG-NF compared with that in the TAU or waitlist group with a moderate effect size (SMD: -0.48). This result suggests the effectiveness of surface EEG-NF when used as a single or add-on therapy against attentional problems in adolescents or young adults with ADHD who only receive routine care. Consistently, a previous meta-analysis comparing surface EEG-NF with non-active controls showed that surface EEG-NF was effective for improving inattention in patients with ADHD (ES: 0.25 assessed by probably blind evaluators and 0.33 by most proximal observers, all *p* < 0.05). Nevertheless, because our subgroup analysis only included two RCTs that enrolled participants with age ranging from 12 to 24 years^16,17^, the finding may not be extrapolated to older adults whose brain plasticity and learning process may be different from those in the younger age group^33^. A possible difference in mechanism underlying the improvement in inattention between children and adults was highlighted by the finding of one of our five included studies that focused on older age groups (18–60 years)^11^. That study demonstrated that clinical improvements in adults were unrelated to the theta-to-beta ratio in EEG^11^, which is believed to be a treatment target for children with ADHD^9^. Further studies are needed to validate our findings and elucidate the impact of age in this setting.

In contrast to the findings of previous meta-analyses that showed consistent positive therapeutic effects of EEG-NF against hyperactivity/impulsivity in children^16,17^. Our subgroup analysis failed to reveal such benefits when compared to participants with TAU or the waitlist patients. The result may partly be explained by the previous finding that the symptoms of hyperactivity/impulsivity are generally less severe in adolescents and adults than those in children^34^. Besides, the small number of studies included in the present meta-analysis (i.e., five) could not exclude possible improvement in hyperactivity/impulsivity associated with the use of surface EEG-NF in adolescents and adults. In addition, the TB protocol, which was the most common treatment approach in our included studies (i.e., four out of the five trials), was originally designed to enhance arousal rather than suppress impulsivity^9^. Therefore, its therapeutic effect may be better reflected by an improvement in arousal-related manifestations (e.g., inattention) than that in hyperactivity/impulsivity. This was supported by the finding of smaller effect sizes associated with EEG-NF treatment against hyperactivity/impulsivity compared to those related to improving inattention in previous meta-analyses^16,17^.

With regards to long-term treatment effects, our study did not find superiority of surface EEG-NF to comparison groups which included both active and non-active treatments. Our result was similar to that from a previous meta-analysis^35^. On the other hand, when focusing on comparison with comparators with non-active treatments, that meta-analysis showed more durable treatment effects associated with surface EEG-NF. However, the availability of only one study with follow-up information on patients receiving non-active interventions in the current meta-analysis precluded our conduction of relevant analysis. Finally, the lack of difference in dropout rate between surface EEG-NF and comparison groups with other interventions suggests their comparable tolerability.

The present meta-analysis had its limitations. First, our results are preliminary because of the inclusion of only five RCTs with a limited number of participants (*n* = 279) and need to be supported by more large-scale clinical trials. Second, despite being the first meta-analysis to investigate the therapeutic impact of surface EEG-NF from a patient’s perspective among adolescents and young adults, our findings may not reflect the improvement in neurocognitive ability or observations from teachers or parents. Third, because most of the included studies did not use double-blinding design, the possibility of subjective placebo effects could not be excluded. Fourth, the confounding effects of medications cannot be excluded because four out of our five included trials did not control medication use in the study arm. Therefore, further studies are needed to elucidate the impacts of medications on the therapeutic effects of surface EEG-NF. Finally, although subgroup analyses focusing on different interventions in the comparison groups would minimize bias in the current study, it was only performed to compare surface EEG-NF with TAU/waitlist because of insufficient number of studies for the conduction of other subgroup analyses. Further studies are warranted to evaluate the differences in therapeutic efficacy between surface EEG-NF and other interventions.

## Conclusion

Focusing on self-reported efficacy of surface EEG-NF, the results of the current meta-analysis support the use of surface EEG-NF for improving inattention in adolescents and young adults, either as single or add-on therapy, despite the small numbers of included trials. Nevertheless, current evidence supporting the therapeutic effect of surface EEG-NF against hyperactivity/impulsivity remains inconclusive. Further large-scale randomized controlled trials focusing on adolescents or adults are warranted to support our findings.

## Supplementary Information

Supplementary Information 1.

Supplementary Information 2.

Supplementary Information 3.
