# Supplementary material for: A pilot meta-analysis on self-reported efficacy of neurofeedback for adolescents and adults with ADHD
Source: Sci Rep. 2022 Jun 15;12:9958. doi: 10.1038/s41598-022-14220-y (PMC9200800; doi:10.1038/s41598-022-14220-y)
Supplement: Supplementary file 1 — Supplementary Information 1. [file 41598_2022_14220_MOESM1_ESM.docx]

**Supplemental Table 1.** Applied keyword and the search result in each database

| Database | Keyword | Filter | Date | Result |
| --- | --- | --- | --- | --- |
| PubMed | (neurofeedback) AND (attention or attention-deficit/hyperactivity disorder or ADHD) | RCT | 2021/08/31 | 88 |
| Embase | (neurofeedback) AND (attention or ADHD) | RCT | 2021/08/31 | 148 |
| ClinicalKey | (neurofeedback) AND (attention or attention-deficit/hyperactivity disorder or ADHD) | N/A | 2021/08/31 | 212 |
| Cochrane CENTRAL | (neurofeedback) AND (attention or attention-deficit/hyperactivity disorder or ADHD) | Trials | 2021/08/31 | 330 |
| ScienceDirect | (neurofeedback) AND (attention or attention-deficit/hyperactivity disorder or ADHD) | Research article | 2021/08/31 | 189 |
| Web of Science | (neurofeedback) AND (attention or attention-deficit/hyperactivity disorder or ADHD) | Article | 2021/08/31 | 485 |
| ClinicalTrials.gov | (ADHD) AND (neurofeedback) | N/A | 2021/08/31 | 27 |

Abbreviations: N/A, not applied; RCT, randomized controlled trials
