# Supplementary material for: A pilot meta-analysis on self-reported efficacy of neurofeedback for adolescents and adults with ADHD
Source: Sci Rep. 2022 Jun 15;12:9958. doi: 10.1038/s41598-022-14220-y (PMC9200800; doi:10.1038/s41598-022-14220-y)
Supplement: Supplementary file 3 — Supplementary Information 3. [file 41598_2022_14220_MOESM3_ESM.docx]

**Supplemental Table 3.** Reasons for study exclusion

| **Reason** | **Number of studies** | **References** |
| --- | --- | --- |
| Not targeted for ADHD patients | 9 | ^1-9^ |
| Not RCT | 9 | ^10-18^ |
| Duplicated sample source | 10 | ^19-28^ |
| Lack of available data for analysis | 7 | ^29-35^ |
| Not EEG neurofeedback | 8 | ^36-43^ |
| Not adequate outcome assessment | 22 | ^44-65^ |

Abbreviations: ADHD, attention-deficit hyperactivity disorder; RCT, randomized controlled trial; EEG neurofeedback, electroencephalogram-based neurofeedback

**References**

1 Morales-Quezada, L. *et al.* Neurofeedback impacts cognition and quality of life in pediatric focal epilepsy: An exploratory randomized double-blinded sham-controlled trial. *Epilepsy & behavior : E&B* **101**, 106570, doi:10.1016/j.yebeh.2019.106570 (2019).

2 Bioulac, S. *et al.* Personalized at-home neurofeedback compared with long-acting methylphenidate in an european non-inferiority randomized trial in children with ADHD. *BMC psychiatry* **19**, 237, doi:10.1186/s12888-019-2218-0 (2019).

3 Jirayucharoensak, S., Israsena, P., Pan-Ngum, S., Hemrungrojn, S. & Maes, M. A game-based neurofeedback training system to enhance cognitive performance in healthy elderly subjects and in patients with amnestic mild cognitive impairment. *Clinical interventions in aging* **14**, 347-360, doi:10.2147/cia.s189047 (2019).

4 Ratcliff, C. G. *et al.* A Randomized Controlled Trial of Brief Mindfulness Meditation for Women Undergoing Stereotactic Breast Biopsy. *Journal of the American College of Radiology : JACR* **16**, 691-699, doi:10.1016/j.jacr.2018.09.009 (2019).

5 Yeo, S. N. *et al.* Effectiveness of a Personalized Brain-Computer Interface System for Cognitive Training in Healthy Elderly: A Randomized Controlled Trial. *Journal of Alzheimer's disease : JAD* **66**, 127-138, doi:10.3233/jad-180450 (2018).

6 Azizi, A., Drikvand, F. M. & Sepahvandi, M. A. Comparison of the Effect of Cognitive Rehabilitation and Neurofeedback on Sustained Attention Among Elementary School Students with Specific Learning Disorder: A Preliminary Randomized Controlled Clinical Trial. *Applied psychophysiology and biofeedback* **43**, 301-307, doi:10.1007/s10484-018-9410-8 (2018).

7 Rostami, R. *et al.* Effects of neurofeedback on the short-term memory and continuous attention of patients with moderate traumatic brain injury: A preliminary randomized controlled clinical trial. *Chinese journal of traumatology = Zhonghua chuang shang za zhi* **20**, 278-282, doi:10.1016/j.cjtee.2016.11.007 (2017).

8 Schabus, M. *et al.* Better than sham? A double-blind placebo-controlled neurofeedback study in primary insomnia. *Brain : a journal of neurology* **140**, 1041-1052, doi:10.1093/brain/awx011 (2017).

9 Keith, J. R., Rapgay, L., Theodore, D., Schwartz, J. M. & Ross, J. L. An assessment of an automated EEG biofeedback system for attention deficits in a substance use disorders residential treatment setting. *Psychology of addictive behaviors : journal of the Society of Psychologists in Addictive Behaviors* **29**, 17-25, doi:10.1037/adb0000016 (2015).

10 Riesco-Matias, P., Yela-Bernabe, J. R., Crego, A. & Sanchez-Zaballos, E. What Do Meta-Analyses Have to Say About the Efficacy of Neurofeedback Applied to Children With ADHD? Review of Previous Meta-Analyses and a New Meta-Analysis. *J Atten Disord* **25**, 473-485, doi:10.1177/1087054718821731 (2021).

11 Van Doren, J. *et al.* Sustained effects of neurofeedback in ADHD: a systematic review and meta-analysis. *Eur Child Adolesc Psychiatry* **28**, 293-305, doi:10.1007/s00787-018-1121-410.1007/s00787-018-1121-4 [pii] (2019).

12 Rossiter, T. The effectiveness of neurofeedback and stimulant drugs in treating AD/HD: part II. Replication. *Applied psychophysiology and biofeedback* **29**, 233-243, doi:10.1007/s10484-004-0383-4 (2004).

13 Fuchs, T., Birbaumer, N., Lutzenberger, W., Gruzelier, J. H. & Kaiser, J. Neurofeedback treatment for attention-deficit/hyperactivity disorder in children: a comparison with methylphenidate. *Applied psychophysiology and biofeedback* **28**, 1-12, doi:10.1023/a:1022353731579 (2003).

14 Ryoo, M. & Son, C. Effects of Neurofeekback Training on EEG, Continuous Performance Task (CPT), and ADHD Symptoms in ADHD-prone College Students. *Journal of Korean Academy of Nursing* **45**, 928-938, doi:10.4040/jkan.2015.45.6.928 (2015).

15 Qian, X. *et al.* Brain-computer-interface-based intervention re-normalizes brain functional network topology in children with attention deficit/hyperactivity disorder. *Translational psychiatry* **8**, 149, doi:10.1038/s41398-018-0213-8 (2018).

16 Pakdaman, F., Irani, F., Tajikzadeh, F. & Jabalkandi, S. A. The efficacy of Ritalin in ADHD children under neurofeedback training. *Neurological sciences : official journal of the Italian Neurological Society and of the Italian Society of Clinical Neurophysiology* **39**, 2071-2078, doi:10.1007/s10072-018-3539-3 (2018).

17 Coben, R., Hammond, D. C. & Arns, M. 19 Channel Z-Score and LORETA Neurofeedback: Does the Evidence Support the Hype? *Applied psychophysiology and biofeedback* **44**, 1-8, doi:10.1007/s10484-018-9420-6 (2019).

18 Heinrich, H., Gevensleben, H., Becker, A. & Rothenberger, A. Effects of neurofeedback on the dysregulation profile in children with ADHD: SCP NF meets SDQ-DP - a retrospective analysis. *Psychological medicine* **50**, 258-263, doi:10.1017/s0033291718004130 (2020).

19 Janssen, T. W. *et al.* A randomized controlled trial into the effects of neurofeedback, methylphenidate, and physical activity on EEG power spectra in children with ADHD. *Journal of child psychology and psychiatry, and allied disciplines* **57**, 633-644, doi:10.1111/jcpp.12517 (2016).

20 Meisel, V., Servera, M., Garcia-Banda, G., Cardo, E. & Moreno, I. Neurofeedback and standard pharmacological intervention in ADHD: a randomized controlled trial with six-month follow-up. *Biol Psychol* **94**, 12-21, doi:10.1016/j.biopsycho.2013.04.015 (2013).

21 Baumeister, S. *et al.* The impact of successful learning of self-regulation on reward processing in children with ADHD using fMRI. *Attention deficit and hyperactivity disorders* **11**, 31-45, doi:10.1007/s12402-018-0269-6 (2019).

22 Beauregard, M. & Lévesque, J. Functional magnetic resonance imaging investigation of the effects of neurofeedback training on the neural bases of selective attention and response inhibition in children with attention-deficit/hyperactivity disorder. *Applied psychophysiology and biofeedback* **31**, 3-20, doi:10.1007/s10484-006-9001-y (2006).

23 Aggensteiner, P. M. *et al.* Slow cortical potentials neurofeedback in children with ADHD: comorbidity, self-regulation and clinical outcomes 6 months after treatment in a multicenter randomized controlled trial. *Eur Child Adolesc Psychiatry* **28**, 1087-1095, doi:10.1007/s00787-018-01271-8 (2019).

24 Steiner, N. J., Frenette, E. C., Rene, K. M., Brennan, R. T. & Perrin, E. C. In-school neurofeedback training for ADHD: sustained improvements from a randomized control trial. *Pediatrics* **133**, 483-492, doi:10.1542/peds.2013-2059 (2014).

25 Vollebregt, M. A., van Dongen-Boomsma, M., Buitelaar, J. K. & Slaats-Willemse, D. Does EEG-neurofeedback improve neurocognitive functioning in children with attention-deficit/hyperactivity disorder? A systematic review and a double-blind placebo-controlled study. *Journal of child psychology and psychiatry, and allied disciplines* **55**, 460-472, doi:10.1111/jcpp.12143 (2014).

26 Liechti, M. D. *et al.* First clinical trial of tomographic neurofeedback in attention-deficit/hyperactivity disorder: evaluation of voluntary cortical control. *Clin Neurophysiol* **123**, 1989-2005, doi:10.1016/j.clinph.2012.03.016 (2012).

27 Bink, M., van Nieuwenhuizen, C., Popma, A., Bongers, I. L. & van Boxtel, G. J. Behavioral effects of neurofeedback in adolescents with ADHD: a randomized controlled trial. *Eur Child Adolesc Psychiatry* **24**, 1035-1048, doi:10.1007/s00787-014-0655-3 (2015).

28 Gevensleben, H. *et al.* Distinct EEG effects related to neurofeedback training in children with ADHD: a randomized controlled trial. *Int J Psychophysiol* **74**, 149-157, doi:10.1016/j.ijpsycho.2009.08.005 (2009).

29 Norouzi, E., Hossieni, F. & Solymani, M. Effects of Neurofeedback Training on Performing Bimanual Coordination In-phase and Anti-phase Patterns in Children with ADHD. *Applied psychophysiology and biofeedback* **43**, 283-292, doi:10.1007/s10484-018-9408-2 (2018).

30 Logemann, H. N., Lansbergen, M. M., Van Os, T. W., Böcker, K. B. & Kenemans, J. L. The effectiveness of EEG-feedback on attention, impulsivity and EEG: a sham feedback controlled study. *Neuroscience letters* **479**, 49-53, doi:10.1016/j.neulet.2010.05.026 (2010).

31 Baumeister, S. *et al.* Neurofeedback Training Effects on Inhibitory Brain Activation in ADHD: A Matter of Learning? *Neuroscience* **378**, 89-99, doi:10.1016/j.neuroscience.2016.09.025 (2018).

32 Döpfner, M. *et al.* ESCAschool study: trial protocol of an adaptive treatment approach for school-age children with ADHD including two randomised trials. *BMC psychiatry* **17**, 269, doi:10.1186/s12888-017-1433-9 (2017).

33 Mohagheghi, A. *et al.* A Randomized Trial of Comparing the Efficacy of Two Neurofeedback Protocols for Treatment of Clinical and Cognitive Symptoms of ADHD: Theta Suppression/Beta Enhancement and Theta Suppression/Alpha Enhancement. *BioMed research international* **2017**, 3513281, doi:10.1155/2017/3513281 (2017).

34 Thompson, L. & Thompson, M. Neurofeedback combined with training in metacognitive strategies: effectiveness in students with ADD. *Applied psychophysiology and biofeedback* **23**, 243-263, doi:10.1023/a:1022213731956 (1998).

35 Holtmann, M., Pniewski, B., Wachtlin, D., Wörz, S. & Strehl, U. Neurofeedback in children with attention-deficit/hyperactivity disorder (ADHD)--a controlled multicenter study of a non-pharmacological treatment approach. *BMC pediatrics* **14**, 202, doi:10.1186/1471-2431-14-202 (2014).

36 Rubia, K. *et al.* Functional connectivity changes associated with fMRI neurofeedback of right inferior frontal cortex in adolescents with ADHD. *NeuroImage* **188**, 43-58, doi:10.1016/j.neuroimage.2018.11.055 (2019).

37 Zilverstand, A. *et al.* fMRI Neurofeedback Training for Increasing Anterior Cingulate Cortex Activation in Adult Attention Deficit Hyperactivity Disorder. An Exploratory Randomized, Single-Blinded Study. *PloS one* **12**, e0170795, doi:10.1371/journal.pone.0170795 (2017).

38 Criaud, M. *et al.* Increased left inferior fronto-striatal activation during error monitoring after fMRI neurofeedback of right inferior frontal cortex in adolescents with attention deficit hyperactivity disorder. *NeuroImage. Clinical* **27**, 102311, doi:10.1016/j.nicl.2020.102311 (2020).

39 Sherwood, M. S. *et al.* Self-directed down-regulation of auditory cortex activity mediated by real-time fMRI neurofeedback augments attentional processes, resting cerebral perfusion, and auditory activation. *NeuroImage* **195**, 475-489, doi:10.1016/j.neuroimage.2019.03.078 (2019).

40 Lam, S. L. *et al.* Neurofunctional and behavioural measures associated with fMRI-neurofeedback learning in adolescents with Attention-Deficit/Hyperactivity Disorder. *NeuroImage. Clinical* **27**, 102291, doi:10.1016/j.nicl.2020.102291 (2020).

41 Takamura, M. *et al.* Antidepressive effect of left dorsolateral prefrontal cortex neurofeedback in patients with major depressive disorder: A preliminary report. *Journal of affective disorders* **271**, 224-227, doi:10.1016/j.jad.2020.03.080 (2020).

42 Kim, H. C. *et al.* Mediation analysis of triple networks revealed functional feature of mindfulness from real-time fMRI neurofeedback. *NeuroImage* **195**, 409-432, doi:10.1016/j.neuroimage.2019.03.066 (2019).

43 Lee, G. J. & Suhr, J. A. Expectancy Effects on Self-Reported Attention-Deficit/Hyperactivity Disorder Symptoms in Simulated Neurofeedback: A Pilot Study. *Archives of clinical neuropsychology : the official journal of the National Academy of Neuropsychologists* **34**, 200-205, doi:10.1093/arclin/acy026 (2019).

44 Sudnawa, K. K. *et al.* Effectiveness of neurofeedback versus medication for attention-deficit/hyperactivity disorder. *Pediatrics international : official journal of the Japan Pediatric Society* **60**, 828-834, doi:10.1111/ped.13641 (2018).

45 Lee, E. J. & Jung, C. H. Additive effects of neurofeedback on the treatment of ADHD: A randomized controlled study. *Asian journal of psychiatry* **25**, 16-21, doi:10.1016/j.ajp.2016.09.002 (2017).

46 Meisel, V., Servera, M., Garcia-Banda, G., Cardo, E. & Moreno, I. Reprint of "Neurofeedback and standard pharmacological intervention in ADHD: a randomized controlled trial with six-month follow-up". *Biol Psychol* **95**, 116-125, doi:10.1016/j.biopsycho.2013.09.009 (2014).

47 Li, L., Yang, L., Zhuo, C. J. & Wang, Y. F. A randomised controlled trial of combined EEG feedback and methylphenidate therapy for the treatment of ADHD. *Swiss medical weekly* **143**, w13838, doi:10.4414/smw.2013.13838 (2013).

48 Ogrim, G. & Hestad, K. A. Effects of neurofeedback versus stimulant medication in attention-deficit/hyperactivity disorder: a randomized pilot study. *Journal of child and adolescent psychopharmacology* **23**, 448-457, doi:10.1089/cap.2012.0090 (2013).

49 Arnold, L. E. *et al.* EEG neurofeedback for ADHD: double-blind sham-controlled randomized pilot feasibility trial. *J Atten Disord* **17**, 410-419, doi:10.1177/1087054712446173 (2013).

50 Bakhshayesh, A. R., Hänsch, S., Wyschkon, A., Rezai, M. J. & Esser, G. Neurofeedback in ADHD: a single-blind randomized controlled trial. *Eur Child Adolesc Psychiatry* **20**, 481-491, doi:10.1007/s00787-011-0208-y (2011).

51 Christiansen, H., Reh, V., Schmidt, M. H. & Rief, W. Slow cortical potential neurofeedback and self-management training in outpatient care for children with ADHD: study protocol and first preliminary results of a randomized controlled trial. *Front Hum Neurosci* **8**, 943, doi:10.3389/fnhum.2014.00943 (2014).

52 Geladé, K. *et al.* A 6-month follow-up of an RCT on behavioral and neurocognitive effects of neurofeedback in children with ADHD. *Eur Child Adolesc Psychiatry* **27**, 581-593, doi:10.1007/s00787-017-1072-1 (2018).

53 Gevensleben, H. *et al.* Is neurofeedback an efficacious treatment for ADHD? A randomised controlled clinical trial. *Journal of child psychology and psychiatry, and allied disciplines* **50**, 780-789, doi:10.1111/j.1469-7610.2008.02033.x (2009).

54 Heinrich, H., Gevensleben, H., Freisleder, F. J., Moll, G. H. & Rothenberger, A. Training of slow cortical potentials in attention-deficit/hyperactivity disorder: evidence for positive behavioral and neurophysiological effects. *Biol Psychiatry* **55**, 772-775, doi:10.1016/j.biopsych.2003.11.013 (2004).

55 Holtmann, M. *et al.* *Specific effects of neurofeedback on impulsivity in ADHD: Evidence from a prospective randomized pilot study*. (2007).

56 Lansbergen, M. M., van Dongen-Boomsma, M., Buitelaar, J. K. & Slaats-Willemse, D. ADHD and EEG-neurofeedback: a double-blind randomized placebo-controlled feasibility study. *Journal of neural transmission (Vienna, Austria : 1996)* **118**, 275-284, doi:10.1007/s00702-010-0524-2 (2011).

57 Lévesque, J., Beauregard, M. & Mensour, B. Effect of neurofeedback training on the neural substrates of selective attention in children with attention-deficit/hyperactivity disorder: a functional magnetic resonance imaging study. *Neuroscience letters* **394**, 216-221, doi:10.1016/j.neulet.2005.10.100 (2006).

58 Lim, C. G. *et al.* A randomized controlled trial of a brain-computer interface based attention training program for ADHD. *PloS one* **14**, e0216225, doi:10.1371/journal.pone.0216225 (2019).

59 Linden, M., Habib, T. & Radojevic, V. A controlled study of the effects of EEG biofeedback on cognition and behavior of children with attention deficit disorder and learning disabilities. *Biofeedback Self Regul* **21**, 35-49, doi:10.1007/bf02214148 (1996).

60 Maurizio, S. *et al.* Comparing tomographic EEG neurofeedback and EMG biofeedback in children with attention-deficit/hyperactivity disorder. *Biol Psychol* **95**, 31-44, doi:10.1016/j.biopsycho.2013.10.008 (2014).

61 Minder, F., Zuberer, A., Brandeis, D. & Drechsler, R. Informant-related effects of neurofeedback and cognitive training in children with ADHD including a waiting control phase: a randomized-controlled trial. *Eur Child Adolesc Psychiatry* **27**, 1055-1066, doi:10.1007/s00787-018-1116-1 (2018).

62 Moreno-García, I., Meneres-Sancho, S., Camacho-Vara de Rey, C. & Servera, M. A Randomized Controlled Trial to Examine the Posttreatment Efficacy of Neurofeedback, Behavior Therapy, and Pharmacology on ADHD Measures. *J Atten Disord* **23**, 374-383, doi:10.1177/1087054717693371 (2019).

63 Steiner, N. J., Frenette, E. C., Rene, K. M., Brennan, R. T. & Perrin, E. C. Neurofeedback and cognitive attention training for children with attention-deficit hyperactivity disorder in schools. *Journal of developmental and behavioral pediatrics : JDBP* **35**, 18-27, doi:10.1097/dbp.0000000000000009 (2014).

64 Strehl, U. *et al.* Neurofeedback of Slow Cortical Potentials in Children with Attention-Deficit/Hyperactivity Disorder: A Multicenter Randomized Trial Controlling for Unspecific Effects. *Front Hum Neurosci* **11**, 135, doi:10.3389/fnhum.2017.00135 (2017).

65 van Dongen-Boomsma, M., Vollebregt, M. A., Slaats-Willemse, D. & Buitelaar, J. K. A randomized placebo-controlled trial of electroencephalographic (EEG) neurofeedback in children with attention-deficit/hyperactivity disorder. *The Journal of clinical psychiatry* **74**, 821-827, doi:10.4088/JCP.12m08321 (2013).
